# Spontaneous hemorrhage of spinal epidural capillary hemangioma resulting in hyperacute neurologic deficit: A case report

**DOI:** 10.1097/MD.0000000000035606

**Published:** 2023-10-20

**Authors:** Marwa Kliea, Mohammad Alsultan, Eyad Chatty, Safaa Qatleesh, Ghassan Hamzeh

**Affiliations:** a Department of Neurology, Al Assad and Al Mouwasat University Hospitals, Damascus University, Faculty of Medicine, Damascus, Syria; b Department of Nephrology, Al Assad and Al Mouwasat University Hospitals, Damascus University, Faculty of Medicine, Damascus, Syria; c Histopathology Department at Al Assad University Hospital, Damascus University, Faculty of Medicine, Damascus, Syria; d Neurology Department at Al Assad University Hospitals, Damascus University, Faculty of Medicine, Damascus, Syria.

**Keywords:** case report, dumbbell-shaped, hyperacute neurologic deficit, spinal cord injury (SCI), spinal epidural capillary hemangioma (SECH)

## Abstract

**Introduction:**

Spinal epidural capillary hemangioma is a very rare variety of tumors, usually with a predilection for the thoracic spine.

**Case presentation:**

A 16-year-old female complained of hyperacute neurologic deficit progressed within hour, which presented by acute paraplegia, and loss of all sensations from her lower limbs up to her breasts. Neurologic exam revealed paralysis of lower limbs (0/5 on both legs) with a flaccid tone, absence of reflexes, weakness of the trunk with sensory level T4, bilateral flexion of plantar reflexes, and loss of sphincters’ controls. Emergent magnetic resonance imaging showed a dumbbell-shaped epidural mass in the posterior aspect of the spinal canal at the T1–T2 level, measuring approximately 1.1 × 4.5 × 1.5 cm in size. The lesion was isointense on T1-weighted, hyperintense on T2-weighted, and a little enhancement after gadolinium administration. The surgery was obtained nearly 16 hours after paralysis, which eradicated the lesion with good hemostasis. Histological examination showed a well-organized vascular tissue that haphazardly arranged and confirmed the diagnosis of capillary hemangioma. Neurological improvement was quickly observed within days after surgery and further complete recovery was achieved 2 months after discharge.

**Conclusion:**

We report an extremely rare case of spinal epidural capillary hemangioma, where acute spontaneous hemorrhage in the lesion resulted in the hyperacute neurologic deficit within an hour. Since these are benign lesions, the immediate surgical intervention results in a very favorable prognosis and is considered the treatment of choice. Also, this case highlighted and rose the question of a better neurologic improvement in younger age patients with spinal cord injury.

## 1. Introduction

Spinal vascular tumors are classified as capillary telangiectasias, cavernous hemangiomas, capillary hemangiomas, arteriovenous malformations, or venous malformations.^[1]^

Hemangiomas of the spine are usually lesions of the vertebral bodies, usually located in the posterior part of the epidural space with a predilection for the thoracic spine.^[1,2]^ Paravertebral extension of the lesion through the intervertebral neural foramina may frequently occur.^[3]^ Most patients present in their fourth to fifth decades of life, with a median age of 49.2 years.^[4]^ There is a slight predilection for females, with a male-to-female ratio of 9:13.^[4]^

Hemangiomas constitute approximately 4% of all epidural tumors and 12% of all intraspinal hemangiomas.^[2]^ Through the literature reports, spinal epidural hemangioma is a very rare variety of tumor, in addition, the capillary type is far rarer than the cavernous type. Approximately 100 cases of cavernous epidural hemangioma compared to only 22 cases of spinal epidural capillary hemangioma (SECH) that were reported previously in the literature.^[4,5]^

According to the literature, magnetic resonance imaging (MRI) with contrast is the diagnostic modality of choice for all spinal cord lesions, including SECH.^[4,6]^ Well-circumscribed margins (dumbbell shape) of the lesion and typically locates in the posterior portion of the spinal canal.^[4,6,7]^ Furthermore, surgical resection is the mainstay treatment for SECH, with a good prognosis, in most cases without recurrence.^[4,8]^

Here we report the 23rd case of capillary hemangioma in a 16-years-old female. The great distinction of this case is that symptoms were hyperacute and developed within an hour. Despite the decompression surgery being obtained nearly 16 hours after paralysis, the neurological improvement was quickly observed within days after surgery and further complete recovery was achieved 2 months after discharge.

## 2. Case presentation

A 16-year-old female was admitted to our neurology department with acute lower limbs paralysis. She had back pain at 2:00 am followed by diffused numbness throughout her lower limbs. Approximately 1 hour thereafter, she could not move her lower limbs, and her back, and she lost all sensations from her lower limbs up to her breasts. She arrived at the hospital after nearly 10 hours after paralysis. There was no traumatic accident and she had no significant past medical history.

On examination, blood pressure was 120/80 mm Hg, heart rate was 85/minutes, respiratory rate was 18/minutes, oxygen saturation was 98% in the air room, and the temperature was 37 °C. Neurological examination revealed paralysis of lower limbs (0/5 on both legs) with a flaccid tone, absence of reflexes, weakness of the trunk with sensory level T4, and bilateral flexion of plantar reflexes. Also, there was a loss of sphincters’ controls where an atonic bladder evacuated 1500 mL after catheter placement.

**Figure 1.**
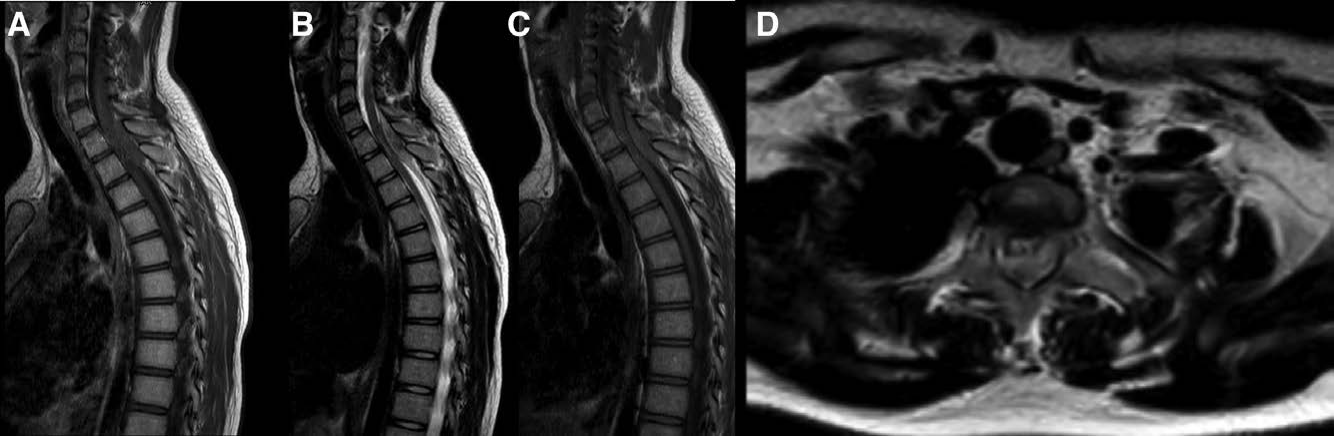
Before the surgery. MRI of the cervical and thoracic spine shows a dumbbell-shaped epidural mass in the posterior aspect compressing the spinal cord at the T1–T2 level. (A) Sagittal T1WI. (B) Sagittal T2WI. (C) Sagittal T1WI with contrast. (D) Axial T2WI. MRI = magnetic resonance imaging.

Emergent MRI was conducted: axial and sagittal T1- and T2-weighted, and post-gadolinium sequences (Fig. 1). MRI of the cervical and thoracic spine showed a well-defined, dumbbell-shaped epidural mass in the posterior aspect of the spinal canal at the T1–T2 level, measuring approximately 1.1 × 4.5 × 1.5 cm in size (Fig. 1). The lesion was isointense on T1-weighted (Fig. 1A), hyperintense on T2-weighted (Fig. 1B), and a little enhancement after gadolinium administration (Fig. 1C). Sagittal (Fig. 1B) and axial T2WI (Fig. 1D) images showed anterior displacement and compression of the spinal cord and the posterior dura layer by the tumor.

The surgery was obtained nearly 16 hours after paralysis under the family and patient acceptance, despite the poor prognosis that was expected by the surgeon. The operation under general anesthesia with middle incision cervical and upper thoracic (C7–T1–T2), showed a hemorrhagic, well-circumscribed, and compressive epidural mass, which was eradicated completely with good hemostasis, and the sample was sent to pathology.

**Figure 2.**
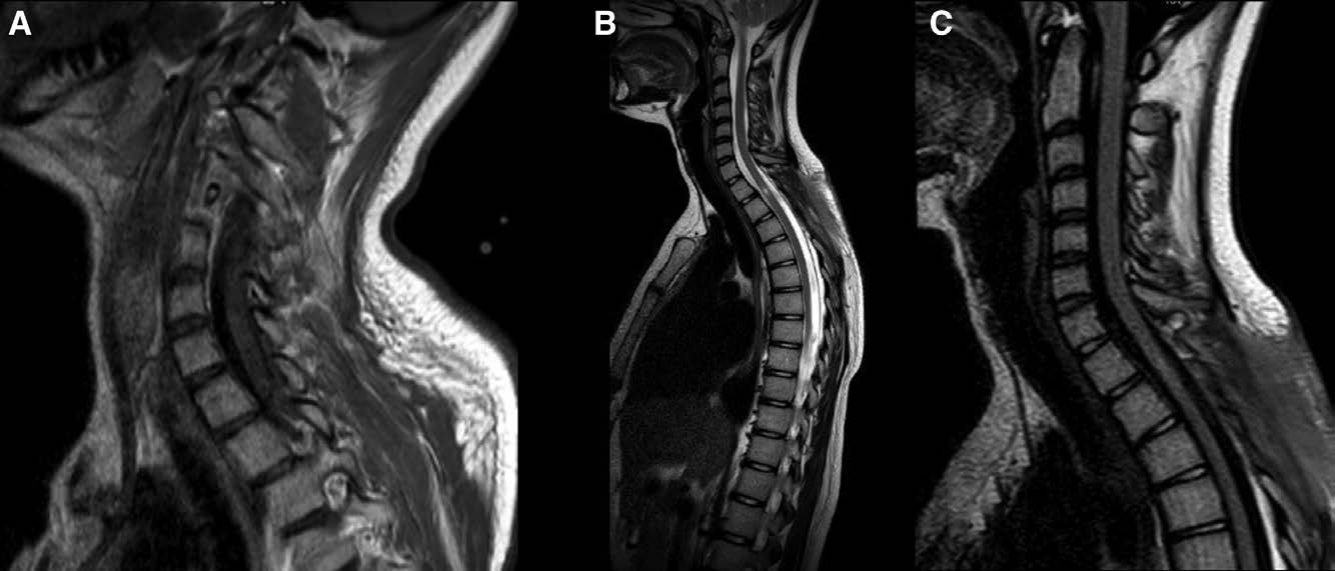
MRI follows up; (A) sagittal T1WI; 24 hours after surgery; shows no mass effect with a drain. (B) Sagittal T2WI and flair (C); 2 months after discharge; show no recurrence of the tumor with a free voiding of CSF. MRI = magnetic resonance imaging.

We continued the conservative management after the surgery with airbed, continuous mobilization, physiotherapy, infection control, and methylprednisolone (MP) 1 g for 5 days followed by tapering doses. subsequent MRI revealed no mass effect with a drain 24 hours after surgery (Fig. 2A) and no recurrence of the tumor with a free voiding of cerebrospinal fluid (CSF) 2 months after discharge (Fig. 2B, C).

**Figure 3.**
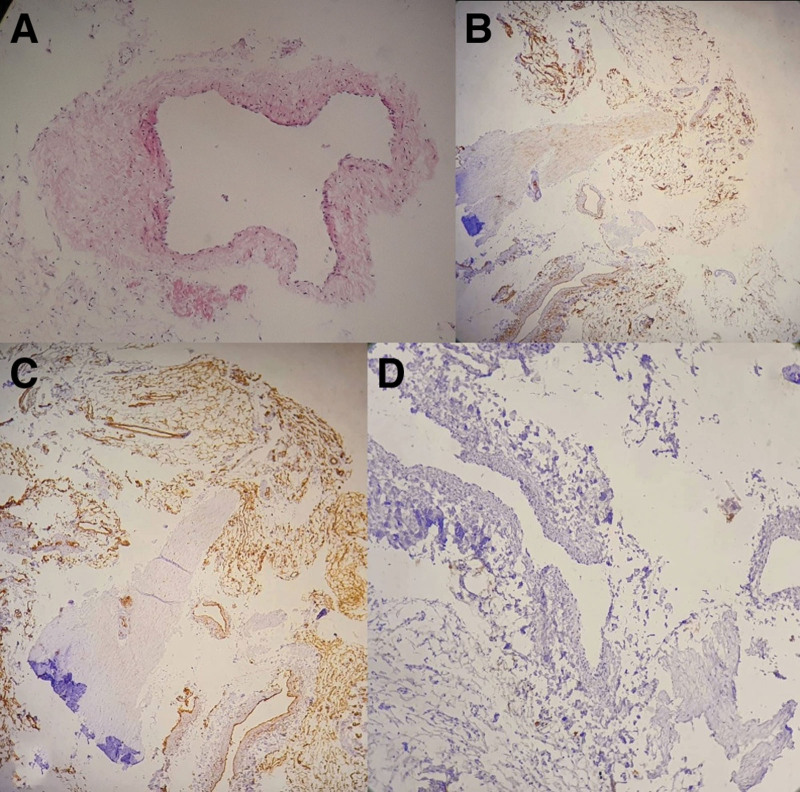
Histopathological figures; showed capillary vessels haphazardly arranged and all vascular channels are lined by regular endothelial cells; (A) PAS stain; shows well-organized vascular vessel. (B) CD34 immunostain; was positive for endothelium (×40). (C) Vimentin immunostain; was positive within normal distribution (×40). (D) Ki67 immunostain; (proliferative index) was negative (×100).

The specimen was fixed by formalin 10% and embedded paraffin and serially sectioned. Routine staining with hematoxylin and eosin was performed (Fig. 3A). Histological examination revealed multifocal nonneoplastic proliferation composed of well-organized vascular tissue composed of small and medium vessels (capillary type) in diameters which haphazardly arranged. All vascular channels were lined by regular endothelial cells without atypical or abnormal mitosis. No large vessels or thick-walled blood vessels such as veins or arteries were noticed. Immunostains were applied, where CD34 (Fig. 3B) was positive for endothelium, vimentin (Fig. 3C) was positive within normal distribution, and Ki67 (Fig. 3D) (proliferative index) was negative. Pathological diagnosis confirmed a capillary hemangioma, that showed only small and middle vessels surrounded by a thin capsule without any large vessels (Fig. 3).

Three days after the procedure, the patient could move her right toes, and the anal sphincter sensation was recovered but not the bladder sphincter. She continued physiotherapy and was discharged after 18 days when the neurologic examination revealed strength in the right leg at 3/5 and 1/5 in the left leg. Two months later the patient returned completely normal, could walk without help, restored the body sensation, and bladder sphincter sensation.

## 3. Discussion

Hemangiomas are congenital vascular malformations, classified according to the predominant vascular morphology into cavernous, capillary, arteriovenous, or venous.^[7]^ Capillary hemangiomas are the most common subtype overall as they occur frequently in the skin and oral mucosa of infants and children.^[7]^ Whereas these tumors are very rare in the spinal cord, the natural history of SECH is not well-defined.^[9]^ SECH on the other hand is exceedingly rare, with only 22 reported cases in the literature.^[4]^

Most patients present with progressive spinal cord syndrome (back pain, myelopathy, or radiculopathy) for several weeks or months due to the slow-growing nature of SECH.^[4]^ Additionally, there were a few cases of SECH that presented with acute spinal cord syndrome due to acute spontaneous or post-traumatic hemorrhage into the lesion.^[1]^ Kidwell et al reported 22 years old male with acute progressive lower extremity weakness and paresthesia after a fall onto his spine. MRI revealed an expanding hematoma over 24 hours.^[10]^ Gencpinar et al also reported a case of a 17-month girl who presented acutely with the inability to walk and restlessness for 1 day without a history of trauma. MRI revealed an extradural dorsal mass at the T3–T7 vertebral level, whereas histopathological examination revealed a capillary hemangioma.^[11]^ Another case by Hakan et al showed a 36 years woman with subacute progressive weakness and numbness for about 2 months during her third trimester. The symptoms progressed more quickly within a week after delivery. MRI showed an epidural mass at the dorsal aspect of T5 and T6 levels compressing the spinal cord and the pathological diagnosis revealed a mixed capillary and cavernous hemangioma.^[2]^

Our patient’s status progressed more severely than in previous reports where she was paralyzed within an hour. Also, there was no previous history of trauma suggesting an acute spontaneous hemorrhage in the lesion that resulted in the patient’s symptoms.

The surgical resection with or without embolization has been advocated, even in the absence of neurological symptoms, to prevent deterioration and permanent neurological deficits if acute spontaneous or post-traumatic hemorrhage into the lesion occurred.^[4,8,12]^

MRI typically demonstrates epidural well-circumscribed (dumbbell shape) mass that locates in the posterior portion of the spinal canal.^[4,6,7]^ The lesion typically presents as an iso- or hypointense lesion on T1-weighted images, hyperintense on T2-weighted images, and homogenous enhancement following gadolinium administration.^[1,6]^ However, these imaging features are not specific to SECH, hence it can be challenging to distinguish from other epidural tumors.^[4,6]^ Moreover, SECH is often misdiagnosed radiologically as being meningiomas and schwannomas because of post-contrast enhancement demonstration of all these types.^[13]^ Many differential diagnoses might be considered such as nonneoplastic lesions; including disc herniation, epidural hematoma, lipomatosis, and abscess, and neoplastic lesions; including meningioma, lymphoma, hemangioblastoma, sarcoma, and even metastases.^[14,15]^

Hence histologic confirmation is necessary in most cases, pathological diagnosis is also necessary to differentiate between hemangiomas and cavernous malformations.^[4,12]^ The latter showed a large number of sinusoidal channels spread in collagenous tissue.^[16]^ By lateral, capillary hemangiomas are composed of thin and irregular capillary vessels en veloped by fibrous stroma or capsule.^[1,12,17]^

Corresponding with previous reports, the SECH was observed in this patient as a dumbbell-shaped epidural mass in the posterior aspect of the spinal canal at the T1–T2 level that compressed the spinal cord. The lesion on MRI was isointense on T1-weighted and hyperintense on T2-weighted with a little enhancement after gadolinium administration. Histological examination aligned with immunostains confirmed haphazardly arranged vascular tissue surrounded by thin capsule.

In the surgical field of spinal cord injury (SCI), there is a question about the ideal time after the trauma to perform the surgical procedure. The studies divide the time until surgery after the injury into 2 categories: “early” and “late.”

A large prospective cohort of the STASCIS (Surgical Timing in Acute Spinal Cord Injury Study) study, found an improvement in the American Spinal Injury scale (AIS scale) in patients undergoing early surgery (<24 hours post-injury) compared to the late surgery (≥24 hours post-injury).^[18]^ Another systematic review and meta-analysis of 16 studies involving 3977 patients with SCI observed significant improvements in motor scores, light touch scores, and sensitivity in the early group (<24 hours) compared with patients in the late group (>24 hours).^[19]^

Recently, a literature review with a meta-analysis of 9 articles by Iunes et al showed a potential neurological improvement with early or even ultra-early surgical decompression (up to 12 hours) in patients with traumatic cervical SCI. On the other hand, reports about the advantage of early decompression when there is a thoracic injury are scarce.^[20]^

The gap in robust data on the beneficial effect of the intervention within 24 hours can be explained as many studies do not specify the area of SCI. Aarabi et al found no neurological benefit in patients with traumatic cervical SCI undergoing decompression within 12 hours of injury, compared with 12 to 24 hours or >24 hours. Meanwhile, the extent of the intramedullary injury was the only significant predicting factor of the improvement in AIS scale.^[21]^

Another systematic review including 14 studies and 1075 patients by Ter Wengel et al investigated the neurological outcome for injuries of the thoracic and thoracolumbar region (T11–L2) between early and late surgery groups. However, the authors did not observe a significant beneficial effect of 24-hour surgical decompression, the rate of AIS scale improvement in the early surgery group was better than in the late surgery group.^[22]^

An important limitation of the studies presented is the lack of homogeneity among patients. Fehlings et al and Cengiz et al observed the average age of patients in the early group was lower than that of those in the late group. This was probably because most surgeons opted for earlier surgical operations in younger patients.^[18,23]^ Also, Sewell et al reviewed that the early decompression group was associated with shorter ICU stay and a lower complication rate but no significant difference in neurological improvement was observed between the 2 groups.^[24]^ However, the author mentioned that neurological recovery was more noticeable in younger patients.^[24]^

Our patient had paraplegia for approximately 16 hours so the surgeon was not excited to do the operation and suspected a bad prognosis. However, the young age of the patient and upon the family’s acceptance, the decision was made to obtain the surgery trying to give a chance for at least a few neurological improvements. Fortunately, the complete recovery was quickly observed between 2 to 3 months after surgery. This corresponds with the previous studies that mentioned the benefit of early decompression (within 24 hours) for SCI. Also, this might suggest a further neurologic recovery in younger age patients and rose the question of a better neurologic improvement in younger age patients with SCI mentioned previously by Sewell et al.

However, the administration of MP was upon the surgeon’s recommendation in our case, there was no rationale for this practice in SCI. Hence, scattered reports of Class III evidence have been published supporting the neuroprotective effect of MP in SCI but these results are likely related to random chance or selection bias.^[25,26]^

Based on the current evidence, high-dose MP treatment does not contribute to better neurologic recoveries and does not demonstrate a significant long-term benefit.^[27,28]^ Clinicians considering MP therapy should bear in mind that the drug is not Food and Drug Administration approved for this indication. Also, the pooled evidence existed that high-dose steroids were associated with harmful effects including wound infection, hyperglycemia, and gastrointestinal hemorrhage.^[29]^

Moreover, we referred to the valuable role of supportive care in the treatment, including physiotherapy and continuous mobilization, to help the patient’s recovery which might be more quickly in younger patients as observed in our patient.

## 4. Conclusion

We report an extremely rare case of SECH that manifested with a hyperacute presentation where acute spontaneous hemorrhage in the lesion resulted in the patient’s symptoms within an hour. Early and prompt diagnosis by MRI is vital and hemangiomas should be considered in the differential diagnosis of epidural lesions with dumbbell-shaped appearance, especially at the thoracic level. Since these are benign lesions, the immediate surgical intervention results in a very favorable prognosis and is considered the treatment of choice. Also, this case highlighted and rose the question of a better neurologic improvement in younger age patients with SCI.

## Author contributions

**Investigation:** Marwa Kliea, Mohammad Alsultan, Eyad Chatty, Safaa Qatleesh.

**Methodology:** Marwa Kliea, Mohammad Alsultan.

**Supervision:** Eyad Chatty, Ghassan Hamzeh.

**Validation:** Marwa Kliea, Mohammad Alsultan.

**Visualization:** Marwa Kliea, Mohammad Alsultan.

**Writing – original draft:** Marwa Kliea, Mohammad Alsultan.

**Writing – review & editing:** Marwa Kliea, Mohammad Alsultan, Ghassan Hamzeh.
